# The relationship between the gastric cancer microbiome and clinicopathological factors: a metagenomic investigation from the 100,000 genomes project and The Cancer Genome Atlas

**DOI:** 10.1007/s10120-025-01588-9

**Published:** 2025-02-17

**Authors:** Mary E. Booth, Henry M. Wood, Mark A. Travis, Genomics England Research Consortium, J. C. Ambrose, p. Arumugam, R. Bevers, M. Bleda, F. Boardman-Pretty, C. R. Boustred, H. Brittain, M. A. Brown, M. J. Caulfield, G. C. Chan, A. Giess, J. N. Griffin, A. Hamblin, S. Henderson, T. J. P. Hubbard, R. Jackson, L. J. Jones, D. Kasperaviciute, M. Kayikci, A. Kousathanas, L. Lahnstein, A. Lakey, S. E. A. Leigh, I. U. S. Leong, F. J. Lopez, F. Maleady-Crowe, M. McEntagart, F. Minneci, J. Mitchell, L. Moutsianas, M. Mueller, N. Murugaesu, A. C. Need, P. O‘Donovan, C. A. Odhams, C. Patch, D. Perez-Gil, M. B. Pereira, J. Pullinger, T. Rahim, A. Rendon, T. Rogers, K. Savage, K. Sawant, R. H. Scott, A. Siddiq, A. Sieghart, S. C. Smith, A. Sosinsky, A. Stuckey, M. Tanguy, A. L. Taylor Tavares, E. R. A. Thomas, S. R. Thompson, A. Tucci, M. J. Welland, E. Williams, K. Witkowska, S. M. Wood, M. Zarowiecki, Phil Quirke, Heike I. Grabsch

**Affiliations:** 1 Division of Pathology & Data Analytics, Leeds Institute of Medical Research at St. James’s, University of Leeds, Leeds, UK; 2 Lydia Becker Institute for Immunology and Inflammation, Wellcome Trust Centre for Cell-Matrix Research, Division of Immunology, Immunity to Infection and Respiratory Medicine, Faculty of Biology, Medicine and Health, Manchester Academic Health Sciences Centre, University of Manchester, Manchester, UK; 3 Genomics England, London, UK; 4 William Harvey Research Institute, Queen Mary University of London, London, EC1M 6BQ UK; 5 Department of Pathology, GROW Research Institute for Oncology and Reproduction, Maastricht University Medical Center+, Maastricht, The Netherlands

**Keywords:** Gastric cancer, Gastrointestinal microbiome, Metagenomics

## Abstract

**Background:**

Findings from previous gastric cancer microbiome studies have been conflicting, potentially due to patient and/or tumor heterogeneity. The intratumoral gastric cancer microbiome and its relationship with clinicopathological variables have not yet been characterized in detail. We hypothesized that variation in gastric cancer microbial abundance, alpha diversity, and composition is related to clinicopathological characteristics.

**Methods:**

Metagenomic analysis of 529 GC samples was performed, including whole exome sequencing data from The Cancer Genome Atlas (TCGA) and whole genome sequencing data from the 100,000 Genomes Project. Microbial abundance, alpha diversity, and composition were compared across patient age, sex, tumor location, geographic origin, pathological depth of invasion, pathological lymph node status, histological phenotype, microsatellite instability status, and TCGA molecular subtype.

**Results:**

Gastric cancer microbiomes resembled previous results, with *Prevotella*, *Selenomonas*, *Stomatobaculum*, *Streptococcus*, *Lactobacillus*, and *Lachnospiraceae* commonly seen across both cohorts. Within the TCGA cohort, microbial abundance and alpha diversity were greater in gastric cancers with microsatellite instability, lower pathological depth of invasion, intestinal-type histology, and those originating from Asia. Microsatellite instability status was associated with microbiome composition in both cohorts. Sex and pathological depth of invasion were associated with microbiome composition in the TCGA cohort.

**Conclusion:**

The intratumoral gastric cancer microbiome appears to differ according to clinicopathological factors. Certain clinicopathological factors associated with favourable outcomes in gastric cancer were observed to be associated with greater microbial abundance and diversity. This highlights the need for further work to understand the underlying biological mechanisms behind the observed microbiome differences and their potential clinical and therapeutic impact.

**Supplementary Information:**

The online version contains supplementary material available at 10.1007/s10120-025-01588-9.

## Introduction

Gastric cancer (GC) is the fifth most common cancer globally and the fifth most common cause of cancer death worldwide. In  2022, there were over 960,000 new cases and 660,175 deaths attributable to GC [1]. *Helicobacter pylori* (*H. pylori*) infection is known to increase the risk of developing GC in some individuals and is the only bacterium identified as an International Agency for Research on Cancer (IARC) class one carcinogen [2, 3]. Increased availability and reduced cost of microbial sequencing has progressed GC microbiome research beyond focusing on single microorganisms towards analysis of whole microbiomes of GC patient cohorts.

Recent studies suggest a lower intratumoral microbial alpha (within sample) diversity in patients with GC, compared to the mucosa of patients with GC precancerous conditions [4–7] or healthy controls [8]. Comparisons of alpha diversity of matched tumor and adjacent tissue suggested increased diversity in GC relative to adjacent tissue, possibly related to the reduced abundance of *H. pylori* in established GC [9–11]. Several studies have compared the GC microbiome composition to the microbiome of normal mucosa or precursor lesions [4–6, 8, 12–14]. The overlap in taxa found more commonly in GC between studies is relatively small. There is, therefore, currently no consensus dysbiotic microbiome associated with GC. GC is a heterogenous disease, with variation in incidence and patient outcomes according to geographical origin, sex, histology, and molecular phenotype [1, 15–18]. However, the contribution of different patient and tumor characteristics to this variation is not known and microbiome research has largely considered GC as a single disease. A better understanding of the potential relationship between patient- and tumor-specific characteristics and the GC microbiome is needed.

In metagenomic approaches, non-human reads are aligned to microbial databases. This approach allows the analysis of large patient populations from sequencing databases, such as The Cancer Genome Atlas (TCGA) [19]. Whole metagenome analysis has the advantage of enabling higher level taxonomic identification to species or even subspecies level than 16S ribosomal RNA sequencing, and deeper microbial coverage than whole exome sequencing.

One rarely considered factor in microbiome analyses, including but not limited to metagenomic analyses, is contaminating microbial DNA related to the material sampling process or the laboratory environment [20–24]. Contamination may distort results and its effect has been shown to be exaggerated in studies of low microbial biomass [25, 26]. Most GC microbiome studies, so far, have not included an *in silico* decontamination processes; this may have contributed to the previously reported conflicting results.

The relationship between the microbiome and selected clinicopathological factors has been explored in a small number of studies [11, 27–29]. These studies have predominantly focused on patients from Asia and have not included thorough decontamination processes.

We hypothesized that GC microbial abundance, alpha diversity, and composition vary according to clinicopathological characteristics. We aimed to characterize the microbiome of GC and explore relationships between the microbiome and clinicopathological features using sequencing data and accompanying clinicopathological data from GC from the 100,000 Genomes Project and TCGA, incorporating a custom *in silico* decontamination process. Through identification of patient- and tumor-specific factors associated with differences in the GC microbiome, we aimed to better understand the role of the microbiome in this heterogenous disease.

## Methods

### Genomes Project data acquisition

Whole genome sequencing data of fresh frozen primary gastric adenocarcinoma and matched blood samples, plus clinical metadata from the 100,000 Genomes Project were accessed within the Genomics England Research Environment [30]. All analyses of Genomics England data were performed within the Genomics England Research Environment.

### TCGA data acquisition

Exome sequencing data of fresh frozen primary gastric adenocarcinoma and matched blood samples plus virtual slide images from the TCGA stomach adenocarcinoma project were obtained from the National Institute of Health National Cancer Institute Genomic Data Commons Data Portal [31]. Basic clinical characteristics were obtained from the University of California Xena TCGA hub, (https://tcga.xenahubs.net) and Liu *et al*. [32]. TCGA GC molecular subtype data were obtained from the TCGA Research Network [19]. For the majority of cases, Lauren histological classification was publicly available [33]; for cases where Lauren classification was not available, the classification was provided by a gastrointestinal histopathologist after reviewing the slide images. All CIBERSORT [34] immune cellular fraction estimates and immune subtypes were obtained from Thorsson [35]. Estimates of six pre-selected immune cells (lymphocytes, neutrophils, macrophages, dendritic cells, eosinophils, and mast cells) and immune subtypes were analysed in exploratory analyses.

### Microsatellite instability (MSI) status and TCGA molecular subtypes

For the TCGA cohort, MSI status was obtained from previously published data [36]. In the present study, MSI-low cases were grouped with microsatellite stable (MSS) cases, since MSS and MSI-low were previously reported to be similar with respect to mutations per Mb (mut/Mb) [37]. The TCGA molecular subtype of the TCGA GC cohort was obtained from the TCGA Research Network classifications [19].

Since MSI status and TCGA molecular subtype data were not available for the 100,000 Genomes Project cohort, MSI status and TCGA molecular subtype were inferred. MSI status was inferred using the number of somatic coding variants (SCV) per sample. Epstein-Barr Virus (EBV) status was determined using sequencing count (virions per human cell), and DNA ploidy was obtained from metadata tables. The TCGA subtype was subsequently inferred, based upon Bass *et al*. [38]. See Online Resource data for flowchart and thresholds used to infer TCGA subtype.

### Metagenomic profiling

Microbiome data were generated from sequencing data using the GATK PathSeq algorithm, aligned against the default PathSeq microbial databases [39]. The PathSeq ‘score’ output was used for microbial sequencing reads, except for the decontamination steps where unambiguously mapping reads were used.

### Decontamination

A modified version of the methodology described by Dohlman [26] was used for *in silico* decontamination. Prevalence was defined as at least two unambiguously mapping reads per taxa per sample. For each species, blood prevalence was compared to tissue prevalence in both the 100,000 Genomes Project and TCGA cohorts. One sided Fisher’s exact test was performed for each species-specific comparison, using a significance threshold of q<0·05. An include-list was created from species more prevalent in tissue than in blood (q<0·05) in the 100,000 Genomes Project or TCGA, where blood prevalence was <20% of samples in both cohorts. For all species with q values ≥0·05 and <0·4 from the TCGA cohort, the literature was reviewed and species identified as inhabitants of the digestive or respiratory tracts were manually added to the include-list. In addition, EBV was manually added to the include-list. Decontaminated datasets for both cohorts for downstream analysis were created by filtering the genus and species reads to include only species present on the include-list. All downstream analysis used only the decontaminated datasets.

## Statistical analyses

Analyses were conducted in R [40] within RStudio [41], using stringr [42], dplyr[43], qvalue [44], and vegan [45] packages. Due to differences in sequencing methodologies and availability of metadata between the two cohorts, analyses were performed in either one or both cohorts depending on data availability and similarities across.

Clinicopathological variables used in analyses included: age, sex, tumor location, geographical origin, pathological depth of invasion (pT), pathological lymph node status (pN), histological phenotype, MSI status, and TCGA molecular subtype.

To evaluate abundance, total microbial count was calculated for each sample. Within the 100,000 Genomes Project cohort, microbial abundance was represented by microbes per human cell and was calculated using the following formula:

$$microbes per human cell= \frac{(microbial reads \div microbial genome size)}{(human reads \div human genome size)}$$, after adjustment of the human genome size for DNA ploidy and tumor cell content. Within the TCGA cohort, microbial abundance represented the sum of microbial sequencing reads. No adjustment for human genome size was made using the TCGA data, since data were derived from exome reads and therefore human reads were not representative of the whole genome due to overrepresentation of exons (relative to intergenic regions).

Shannon index [46] was calculated for each sample as a measure of alpha diversity. Wilcoxon and Kruskal–Wallis tests were applied for comparisons of categorical variables. Spearman’s rank correlation coefficient was calculated for correlation analyses. Permutational multivariate analysis of variance (PERMANOVA, Adonis), using Bray-Curtis dissimilarity index, [47] was used to analyze beta diversity in species between subgroups. The PERMANOVA analysis considered clinicopathological variables with over 85% completeness. To avoid overlapping variables, TCGA molecular subtype was not included since it is a composite variable which includes MSI status, which was already included in PERMANOVA analysis. Samples with no taxa were removed prior to PERMANOVA analysis. Variables significant at beta diversity PERMANOVA analysis were included in analysis of differential abundance according to clinicopathological variables. Multivariable Association Discovery in Population-scale Meta-omics Studies (MaAsLin2) [48] was used to perform differential abundance analyses.

## Results

Eighty nine tumor samples from patients with gastric or gastro-oesophageal junction adenocarcinoma (GC) were identified in the 100,000 Genomes Project database. All 89 GC samples had whole genome sequencing data for tumor and blood which were used to generate microbial sequencing data. TCGA microbial sequencing data were generated from whole exome sequencing data from 441 tumor samples and 396 matched blood samples from the TCGA GC cohort. Histological review of available slides from the TCGA GC cohort identified one sample as squamous cell carcinoma, therefore, this case was removed from subsequent analysis. The distribution of clinicopathological features within both the 100,000 Genomes Project and TCGA cohorts can be found in Table 1.

**Table 1:** Clinicopathological characteristics of samples used within this study, from 100,000 Genomes Project and TCGA cohorts

Characteristic	100,000 Genomes Project – gastro-oesophageal cancer total *n* = 89 n (%)	TCGA – gastric cancer total *n* = 440 n (%)
Age, median (interquartile range)	69 (62-77)	67 (58-73)
Unknown	0 (0)	5 (1)
*Sex*		
Male	69 (78)	283 (64)
Female	20 (23)	157 (36)
*Tumor location*		
Cardia	27 (30)	90 (20)
Non-cardia	44 (49)	280 (64)
Unknown	18 (20)	70 (16)
*Geographic origin*		
Asia	0 (0)	69 (15)
Not Asia	89 (100)	314 (71)
Unknown	0 (0)	57 (13)
*Pathological depth of invasion (pT)*		
1	8 (9)	23 (5)
2	11 (12)	93 (21)
3	32 (36)	198 (45)
4	29 (33)	117 (27)
Unknown	9 (10)	9 (2)
*Pathological lymph node status (pN)*		
0	20 (23)	131 (30)
1	25 (28)	117 (27)
2	22 (25)	86 (20)
3	13 (15)	88 (20)
Unknown	9 (10)	18 (4)
*Histological phenotype*		
Diffuse	10 (11)	76 (17)
Intestinal	23 (26)	278 (63)
Mixed	2 (2)	21 (4)
Mucinous	0 (0)	19 (4)
Unknown	54 (61)	46 (10)
*MSI status*		
MSS	81(91)*	308 (70)
MSI	8(9)*	75 (17)
Unknown	0 (0)	57 (13)
*TCGA subgroup*		
EBV	3 (3)*	26 (6)
MSl	8 (9)*	64 (15)
CIN	35 (39)*	145 (33)
GS	43 (48)*	58 (13)
Unknown	0 (0)*	147 (33)
*Immune subtype*^†^		
C1	0 (0)	129 (31)
C2	0 (0)	209 (51)
C3	0 (0)	35 (9)
C4	0 (0)	9 (2)
C5	0 (0)	0 (0)
C6	0 (0)	7 (2)
Unknown	89 (100)	51 (12)

*CIN*, chromosomal instability; *EBV*, Epstein-Barr virus-positive; *GS*, genomically stable; *MSI*, microsatellite instability; *MSS*, microsatellite stabile; *n*, number

^*^Inferred as described in methods

^†^Immune subtype according to Thorsson *et al.* available for TCGA cohort only

### MSI status and TCGA molecular subtypes

MSI status was available for 383 (87%) TCGA GC from previously published data [36]. MSI data were not directly available for the 100,000 Genomes Project data and an inferred MSI status was used for analysis (Online Resource Figure 1): samples where SCV ≥20 mut/Mb were inferred as MSI; samples where SCV <20 mut/Mb were inferred as MSS.

TCGA molecular subtype status was available for 293/440 (67%) GC from the TCGA cohort. Within the 100,000 Genomes Project, thresholds used to determine inferred TCGA molecular subtype were: EBV count 1x10^−3^ per human cell; SCV 20mut/Mb; and DNA ploidy 2·5 (Online Resource Figure 1). The distribution of samples according to inferred MSI and TCGA molecular subtype is shown in Table 1.

### Decontamination

**Fig. 1:**
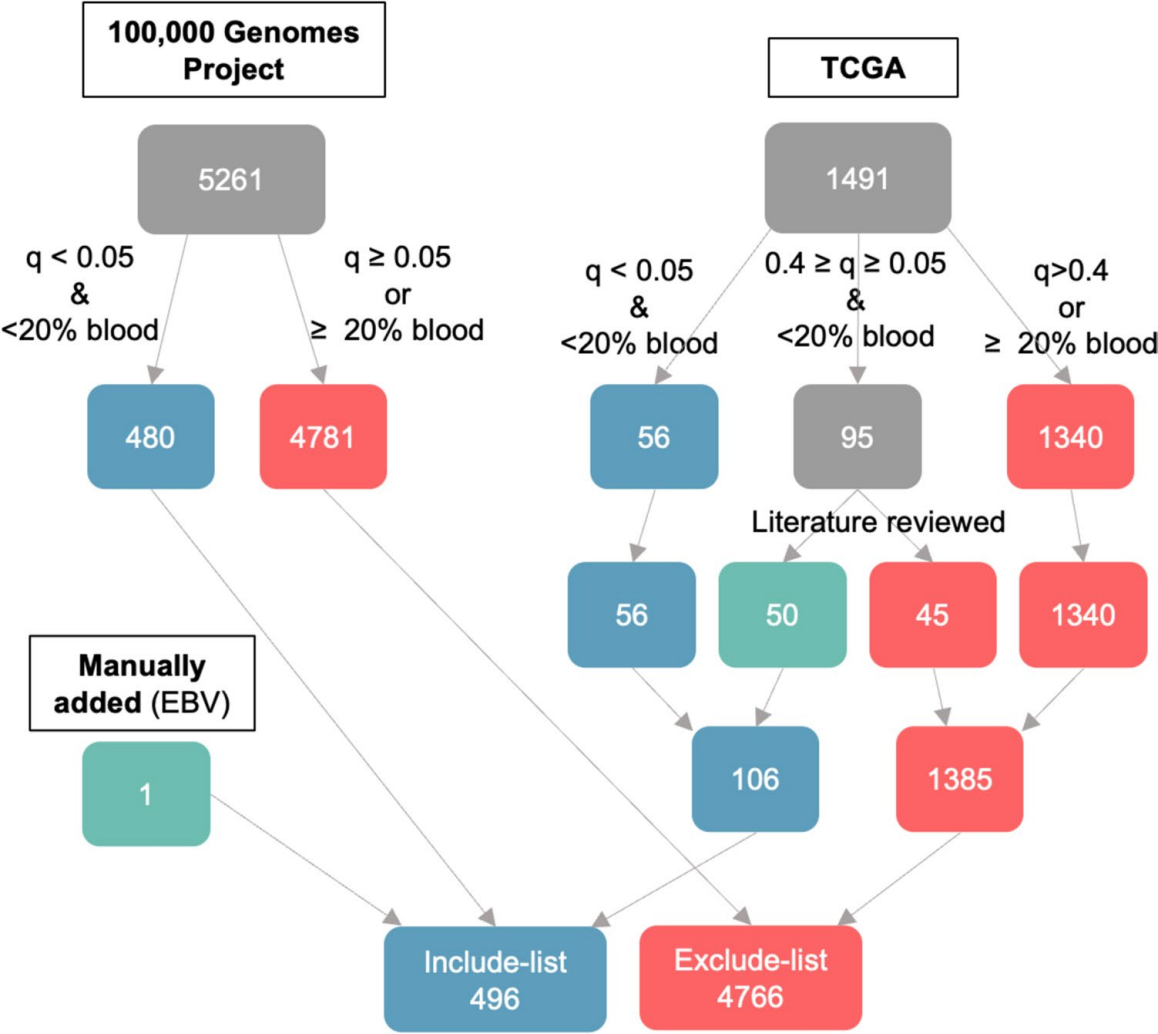
Overview of the decontamination process. Flow chart to demonstrate categorisation of species into the study include- or exclude-lists, using both 100,000 Genomes Project and TCGA species-level data. The numbers in the final include- and exclude-lists total less than the species upstream, as some species were prevalent within both datasets. *EBV*, Epstein-Barr Virus; *TCGA*, The Cancer Genome Atlas

Prior to decontamination, 5261 species from 946 genera were present across at least one sample from samples of the 100,000 Genomes Project cohort; 1491 species from 374 genera were prevalent across at least one sample from the TCGA cohort. 480 species were identified as tissue-resident from the 100,000 Genomes Project cohort and 106 species (comprising of 56 species where q<0·05 and additional 50 species where q<0·4 and identified as inhabitants of digestive or respiratory tracts) were identified as tissue-resident from TCGA cohort. None of the species identified as tissue-resident from only one cohort had a blood prevalence ≥20% in either cohort. EBV was not statistically assigned to either list due to its low prevalence in the 100,000 Genomes Project cohort (4/89 tumor samples; 0/89 blood samples) and absence within the TCGA cohort (0/441 tumor samples; 0/396 blood samples) and was manually added to the include-list. The final include-list consisted of 496 species from 105 genera. Figure 1 illustrates the decontamination process performed to generate the species include-list.

The decontaminated datasets represented 53 and 78% of the original microbial content for the 100,000 Genomes Project and TCGA, respectively. For the TCGA cohort, 135/440 (31%) of samples had no taxa after the decontamination process, whereas all 100,000 Genomes Project samples had taxa remaining after decontamination.

### Taxonomic composition

Following decontamination, six genera (*Prevotella, Selenomonas, Stomatobaculum, Streptococcus, Lactobacillus*, and *Lachnospiraceae*) were found to be in the 10 top abundant genera in both cohorts. Two species (*Stomatobaculum longum* and *Lachnospiraceae bacterium oral taxon 082*) were common to the 10 top abundant species within the two cohorts. Many samples from the TCGA cohort had no or very low microbial abundance following the decontamination process. There was considerable variation in microbial composition within both cohorts. Heatmaps for the most abundant genera and species are shown in Online Resource Figures 3 and 4.

### Abundance and alpha diversity

Within the 100,000 Genomes Project cohort, no statistically significant relationships were identified between the analysed clinicopathological variables and either microbial abundance or Shannon index. However, when the inferred MSI status of the 100,000 Genomes Project GC cohort was examined in relation to microbial abundance, MSI samples tended to have greater microbial abundance than MSS samples (*p* = 0·061). However, no relationship was identified between MSI status and Shannon index.

**Fig. 2:**
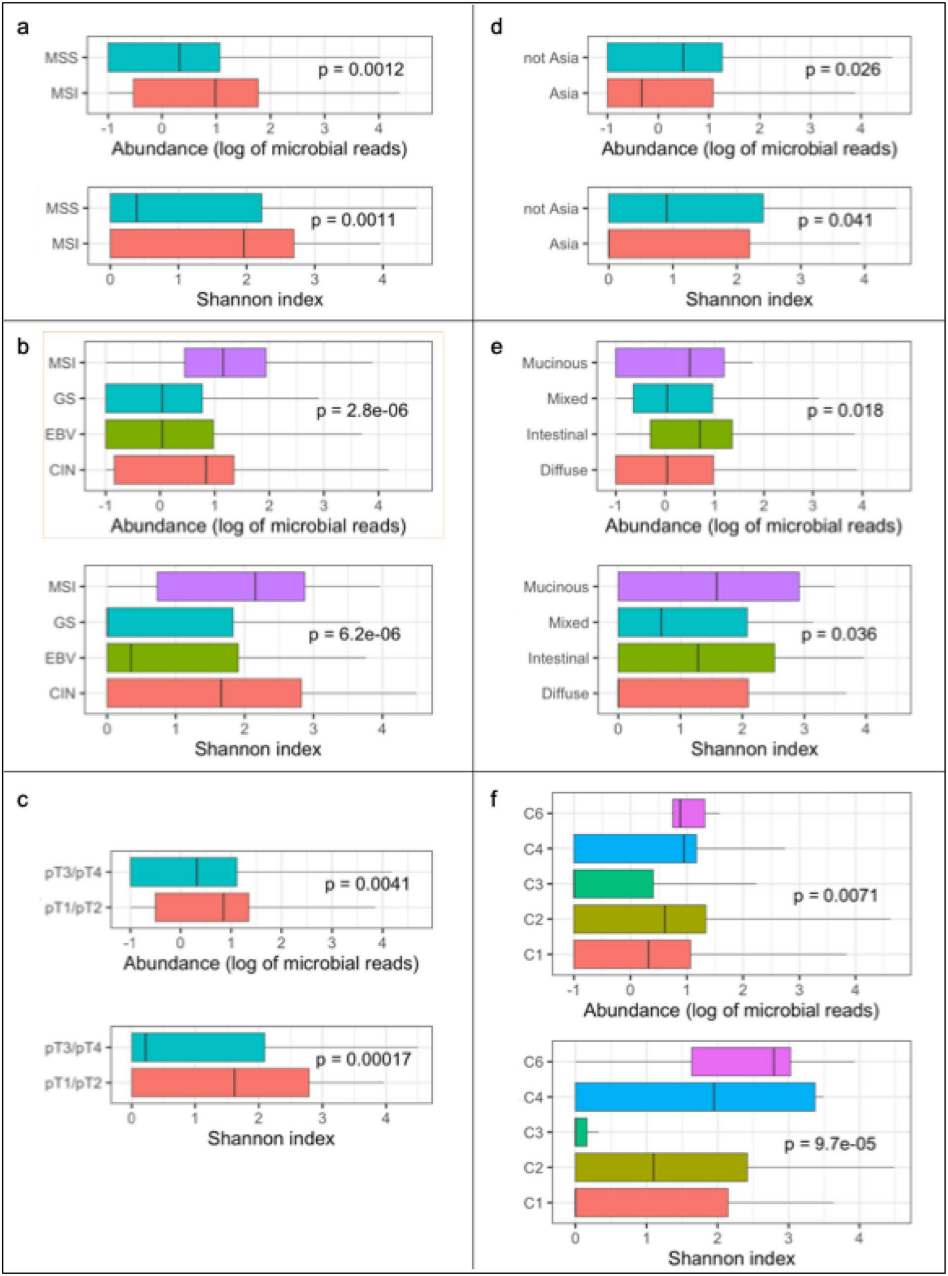
Boxplots of microbial abundance and Shannon index of TCGA samples according to **a** MSI status *n* = 383, **b** TCGA subtype, *n* = 293, **c** pathological depth of invasion (pT), *n* = 431, **d** geography, *n* = 383, **e** histological subtype, and **f** immune subtype. *CIN*, chromosomal instability; *EBV*, Epstein-Barr virus-positive; *GS*, genomically stable; *MSI*, microsatellite instability; *MSS*, microsatellite stable. *p* values represent Wilcoxon and Kruskal–Wallis statistics for comparisons of two variables and more than two variables, respectively

In the TCGA cohort, MSI GC was associated with greater microbial abundance (*p* = 0·001) and Shannon index (*p* = 0·001) than MSS GC (Fig. 2a). Within the TCGA cohort, TCGA molecular subtype was associated with microbial abundance (*p* < 0·001) and Shannon index (*p* < 0·001). The boxplots (Fig. 2b) demonstrate highest microbial abundance and Shannon index in the MSI subtype, and lowest abundance and Shannon index in the genomically stable subtype. Within the TCGA cohort, lower pT category (pT1 and pT2) was associated with both, greater microbial abundance (*p* = 0·004), and greater Shannon index (*p* < 0·001) (Fig. 2c). GC from Asia had lower microbial abundance (*p* = 0·03) and lower Shannon index (*p* = 0·04) than samples not from Asia (Fig. 2d). Histological phenotype was associated with microbial abundance (*p* = 0·02) and Shannon index (*p* = 0·04) whereby mucinous GC and intestinal-type GC had greater microbial abundance than diffuse-type GC (Fig. 2e).

Sex, age, tumor location (cardia versus non-cardia), and pN category were not related to microbial abundance or Shannon index in either cohort.

Immune cellular fraction estimates and immune subtypes data generated from CIBERSORT [34] were available for 389/440 (88%) TCGA GC. Estimates of six immune cell types (lymphocytes, neutrophils, macrophages, dendritic cells, eosinophils, and mast cells) were plotted against both total microbial count and Shannon index. No notable relationships were identified between any of the six immune cell types and either abundance or Shannon index. Immune subtype (C1–C6, according to Thorsson et al.) [35], was associated with a statistically significant difference in both microbial abundance (*p* = 0·007) and Shannon index (*p* < 0·001). The boxplots (Fig. 2f) demonstrate highest microbial abundance and Shannon index in samples belonging to the C4 and C6 subtype, although these only represented 4% of analyzed samples.

## Beta diversity

The PERMANOVA analyses considered variables with over 85% completeness, in a pre-defined order of: geographical origin (TCGA only), age, sex, histological phenotype (TCGA only), MSI status, pT category, and pN category. Removal of samples with missing variables (both cohorts) and no taxa (TCGA only), resulted in 80 samples from the 100,000 Genomes Project and 240 samples from TCGA, available for PERMANOVA analysis. The analysis indicated an association between MSI status and microbial composition within both cohorts. In addition, sex and pT category were associated with differences in microbial composition within the TCGA cohort (Table 2).

**Table 2:** Permutational multivariate analysis of variance (PERMANOVA) for intratumoral species within 100,000 Genomes Project and TCGA

	100,000 Genomes Project (*n* = 80)		TCGA (*n* = 240)	
	R^2^	*P* value	R^2^	*P* value
Geography	–	–	0·00592	0·1246
Sex	0·01415	0·2184	0·00845	0·0237*
Age	0·01383	0·2634	0·00716	0·0559
MSI ^**†**^	0·01727	0·0360*	0·00740	0·0455*
Histology	–	–	0·01448	0·2042
pT	0·01391	0·386	0·00776	0·0363*
pN	0·01306	0·308	0·00337	0·6058
Residuals	0·92777		0·94546	–

*R2*, the proportion of the variance in the microbiome explained by each variable; *MSI* microsatellite instability-high versus non high; *pT*, pathological depth of invasion (pT1/pT2 versus pT3/pT4); *pN*, pathological lymph node status (pN0 versus >pN0)

^*^statistically significant (*p* < 0·05)

^†^Inferred MSI status for 100,000 Genomes Project cohort

**Fig. 3:**
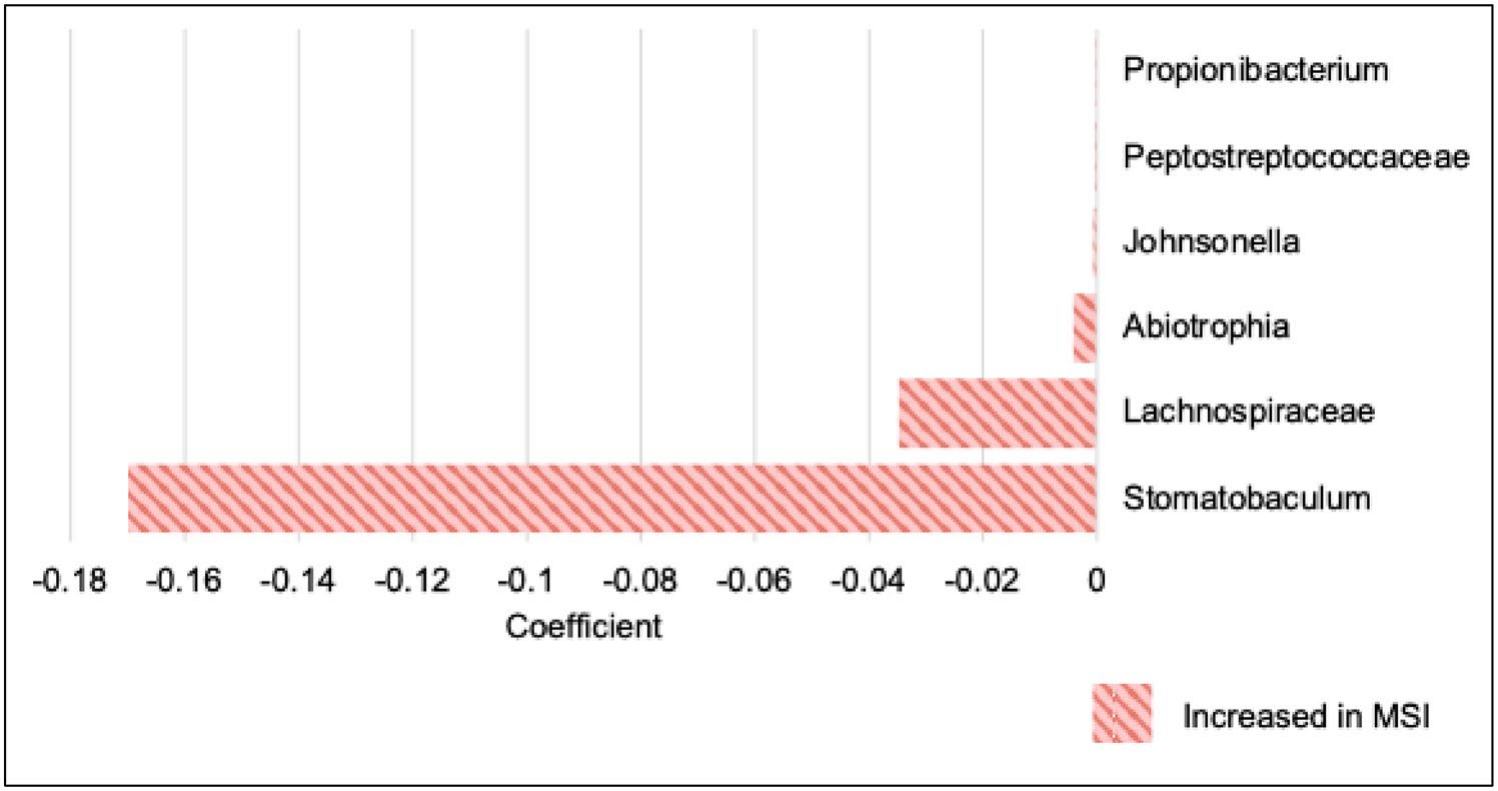
Association of specific genera within the 100,000 Genomes Project samples, according to inferred MSI status by Multivariable Association Discovery in Population-scale Meta-omics Studies (MaAsLin2) (*n* = 89). MaAsLin2 coefficient (effect size) according to MSI status. Red indicates genera enriched in MSI (versus MSS). *MSI*, microsatellite instability; *MSS*, microsatellite stable

In the 100,000 Genomes Project cohort, MaAsLin2 analysis detected 12 species and six genera with statistically significant differential abundance between inferred MSI and MSS inferred subtypes. Figure 3 shows such differential abundances at genus level.

**Fig. 4:**
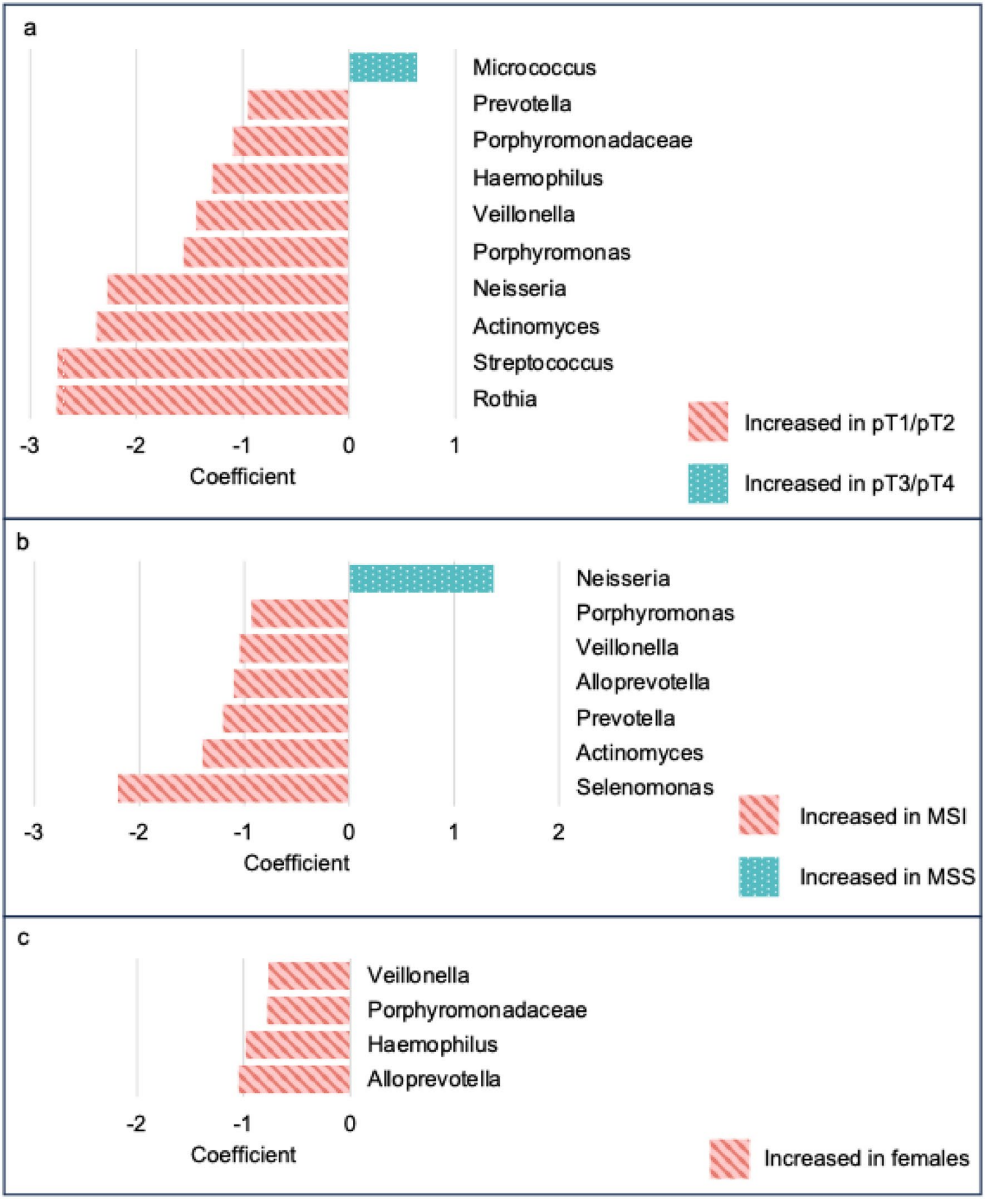
Association of specific genera within TCGA samples, according to pT category, MSI status, and sex by Multivariable Association Discovery in Population-scale Meta-omics Studies (MaAsLin2) (*n* = 375). **a** MaAsLin2 coefficient (relative effect size) according to pT category. Red indicates genera enriched in pT1/pT2; green indicates genera enriched in pT3/pT4. **b** MaAsLin2 coefficient according to MSI status. Red indicates genera enriched in MSI; green indicates genera enriched in MSS. **c** MaAsLin2 coefficient according to sex. Red indicates genera enriched in females (versus males). *MSI*, microsatellite instability; *MSS*, microsatellite stable; *pT*, pathological depth of invasion

In the TCGA cohort, MaAsLin2 analysis identified 45 species and 12 genera differentially abundant across sex, MSI status and pT category. Differences at genus level are shown in Fig. 4. All differentially abundant taxa according to sex were found more commonly in males than in females. All differentially abundant taxa according to pT category were found more commonly in pT1/pT2 GC compared to pT3/pT4 GC, except for the *Micrococcus* genus and *Micrococcus aloeverae* species, which were found in greater abundance in pT3/pT4 GC. All differentially abundant taxa according to MSI status were found more commonly in MSI GC compared to MSS GC, except for the *Neisseria* genus, which was found in greater abundance in MSS GC. Of note, none of the species or genera identified as differentially abundant according to MSI status within the 100,000 Genomes Project were differentially abundant according to MSI status on multivariate analysis in the TCGA cohort.

## Discussion

We explored the relationship between patient- and tumor- specific factors and the GC microbiome. Here, we present the results from an exploratory study of the intratumoral GC microbiome in a total of 529 GC patients, analyzing whole genome sequencing data from the 100,000 Genomes Project and whole exome sequencing data from TCGA. This is the largest GC study to date to use whole genome and whole exome sequencing data to characterize the clinicopathological features associated with the GC microbiome. We identified associations of potential clinical importance through identifying relationships between clinicopathological features and microbial abundance, alpha diversity, and beta diversity.

Within the present study, we further developed the decontamination process as initially described by Dohlman [26], incorporating two separate databases to maximize the number of genuine tumor taxa and minimize contamination. Decontamination is infrequently performed in GC studies; however, the high proportion of the total signal and number of taxa removed emphasize its importance in this setting. This has been previously demonstrated to be particularly important within other low-biomass studies [49, 50]. Furthermore, it has been demonstrated that upstream errors in decontamination processes can have large downstream effect on study results [51]. Eisenhofer *et al*. [49] and Salter *et al*. [50] suggested an exclude-list approach to decontamination, however, this may result in contaminants being missed (and subsequently being analysed within the dataset). The include-list approach suggested by Dohlman *et al*. was validated by comparing against 16S rRNA amplicon sequencing of matched fresh colorectal cancer tissue, resulting in the absence of putative contaminants; this method has subsequently been used in an analysis of over 2000 colorectal cancers [52]. The include-list approach by Dohlman *et al*. may still result in the exclusion of important taxa, as demonstrated by *Escherichia coli* which did not initially meet the criteria for tissue-resident bacterium in The Cancer Microbiome Atlas analysis, resulting in some further manual curation [26]. These studies collectively guided the two-step decontamination approach taken in the present study of low biomass tissue; first a statistical algorithm was applied to exclude known and unknown contaminants, then borderline taxa were reviewed and added to the include-list where biological evidence was sufficient. This is the first study to systematically analyse the relationship between GC clinicopathological characteristics and the GC microbiome using data from patients across the globe using an *in silico* decontamination process.

We found that microbiome abundance, diversity, and composition differ in GC in relation to MSI status of the tumor. Consistent with our findings in GC, MSI status has also been associated with microbial changes in colorectal cancer [53–57]. A recent study by Byrd *et al.* has also investigated the relationship between MSI status and the microbiome within the TCGA cohort [29]. In line with our findings, this study also found an association between intratumoral microbes and MSI status across stomach, colorectal, and endometrial cancers. The methodology used to generate the microbiome abundance data from whole transcriptome and whole genome sequencing data in this study has since been identified as flawed, although to what extent that affects the validity of this paper by Byrd *et al.* is unclear [51, 58]. Whilst the extent to which the relationship between MSI status and the microbiome is associative or causative is uncertain, it is possible that the local tumor environment of MSI GC somehow facilitates a more abundant, more diverse microbiome.

In addition to MSI status, our results from the TCGA analysis suggest greater microbial abundance and alpha diversity in lower pT category GC, as well as a difference in microbial composition between pT1/pT2 and pT3/pT4 GC. These findings may reflect a difference in host environment in that fewer taxa, or only certain specific taxa, are able to invade beyond the muscle wall. These findings are consistent with those of a study investigating only Asian patients [27], where the microbial composition differed according to stage. This study did not identify alpha diversity differences according to stage, which may indicate that changes are more related to the pT classification than overall staging, which incorporates pT and pN classification, as well as the presence or absence of distant metastasis. Molecular differences according to tumor depth have previously been reported in GC [59, 60]; thus, when considering this in the context of our findings, one could speculate that spatial differences in tumor microenvironment influence the local microbiome. Alternatively, a more abundant, more diverse microbiome may be protective against greater tumor invasion, potentially through increased local immune surveillance.

The increased microbial abundance and alpha diversity seen in intestinal-type GC compared to diffuse-type GC may result from differences in the tumor microenvironment. In line with our findings, lower alpha diversity was observed in patients with diffuse-type GC relative to intestinal-type GC, in a study of 64 GC samples from Lithuanian patients [28]. Previous studies in GC have demonstrated evidence of molecular differences [19, 33], as well as differences in the ratio of tumor to stroma [61], according to histological phenotype. Existing data from a single-cell RNA sequencing analysis of GC is suggestive of differences in the proportions of plasma cells and KLF2-expressing epithelial cells between diffuse- and intestinal-type GC [62]. It is possible that molecular variability between diffuse-type and intestinal-type GC could result in differences in microbial abundance and alpha diversity.

We decided to further explore a potential relationship between the GC microbiome and immune cells in the tumor microenvironment, using CIBERSORT data from TCGA, to try to further understand the observed relationships between the microbiome and clinicopathological characteristics including MSI status, pT category, and histological phenotype. The immune subtypes associated with greater microbial abundance and Shannon diversity (C4 and C6) represented the lymphocyte depleted and TGF-b dominant subtypes, respectively, although only representing 4% of analyzed samples. Our investigation of immune cells with microbial abundance and alpha diversity was not able to provide further insight on this, possibly due to the reliance on whole tumor immune data, which was not spatially orientated. Further investigation of the relationship between the GC microbiome and local immune cells is warranted and spatial techniques should be considered in such investigations.

Notably, MSI (versus MSS) GC, low (versus high) pT category, and intestinal (versus diffuse) histological phenotype—all of which have been observed to have greater microbial diversity and abundance within the present study—are generally recognized to be good prognostic factors in GC [63–66]. In general, higher microbial diversity is thought to be associated with improved health outcomes, including in [67], but not limited to [68], patients with cancer. Considered in this context, our own observations of increased microbial diversity in such “good prognosis” subgroups (MSI, low pT and intestinal histology) warrants further investigation of the role of microbial abundance and diversity in GC behaviour—in particular, whether and through what mechanisms increased microbial diversity may contribute to the improved outcomes observed in these good prognosis groups. If results from future studies support the hypothesis that greater GC microbial abundance and diversity results in superior outcomes, this may inform development of therapeutic investigations to increase microbial abundance and diversity, with the aim of improving GC outcomes.

Our study had some limitations. The 100,000 Genomes Project Cohort had small sample numbers (89) whilst the TCGA cohort was based on exome data, which has a limited ability to facilitate detection of microbiome data in the original sample. Recent work [51] has demonstrated that microbiome data from low biomass exome data can be challenging and easy to over-interpret. While gastric samples do not have the extremely low (or absent) non-human content of other tissue types, care should be taken in analysing samples such as the TCGA cohort. In addition, the geographical and racial composition of the two cohorts differed, with the 100,000 Genomes Project including only individuals from England and TCGA including individuals from across the world, including at least 15% from Asia. Whilst no association was detected within the present study between geography and microbial abundance, alpha diversity, or composition, geography may still account for some of the differences in microbiome composition between the two cohorts. *H. pylori* prevalence varies considerably with geographic location, with higher rates of infection in Asian countries than in Western Europe and the United States [69]. *H. pylori* may influence the gastric mucosal microbiome in both neoplastic [70–72] and non-neoplastic [70, 72, 73] stomachs. Therefore, it is possible that the differences in microbial composition detected between cohorts are related to the geographical heterogeneity that may not have been captured by PERMANOVA analysis due to the over-simplification of geography (i.e., Asia versus not-Asia). Furthermore, the availability of metadata differed between the two cohorts, limiting the investigation to a purely exploratory analysis.

In conclusion, using two separate sequencing databases, we identified microbiome differences in relation to depth of tumor invasion, histological phenotype, and molecular characteristics such as presence of MSI. Our findings further reinforce the notion that the relationship between the GC microbiome and clinicopathological variables is multifactorial and, as such, should be considered when planning, conducting, and interpreting the results from investigational clinical studies. Future work should focus on 1) further functional studies to increase the level of understanding regarding how the local microenvironment may affect and be affected by the tumor microbiome, 2) how the microbiome may affect GC phenotype, and 3) whether ultimately microbiome manipulation could affect outcomes of patients with GC and/or response to specific therapeutic interventions. This study collated 529 GC microbiomes; using this large sample size, we were able to consolidate the findings of previous studies [26, 74, 75] and conduct an evaluation of the relationship between microbial differences and patient and tumor characteristics. The findings of this study underline the potential clinical importance of the GC microbiome and provide a strong rationale for further investigations to improve the depth of understanding in this area.

## Supplementary Information

Below is the link to the electronic supplementary material.

Supplementary file1 (PDF 1017 kb)
