# Supplementary material for: The relationship between the gastric cancer microbiome and clinicopathological factors: a metagenomic investigation from the 100,000 genomes project and The Cancer Genome Atlas
Source: Gastric Cancer. 2025 Feb 17;28(3):358–71. doi: 10.1007/s10120-025-01588-9 (PMC11993446; doi:10.1007/s10120-025-01588-9)
Supplement: Supplementary file 1 — Supplementary file1 (PDF 1017 kb) [file 10120_2025_1588_MOESM1_ESM.pdf]

Online Resource Table 1. Table of species present on study include list following decontamination process.

Species	Cohort
Abiotrophia_defectiva	GE only
Abiotrophia_sp._HMSC24B09	GE only
Achromobacter_insuavis	GE only
Achromobacter_ruhlandii	GE only
Achromobacter_sp.	GE only
Acidovorax_sp._KKS102	GE only
Acidovorax_temperans	GE only
Acinetobacter_baumannii	GE only
Acinetobacter_bohemicus	GE only
Acinetobacter_guillouiae	GE only
Acinetobacter_johnsonii	GE only
Acinetobacter_sp._BMW17	GE only
Acinetobacter_sp._CIP_64.7	GE only
Acinetobacter_sp._ETR1	GE only
Acinetobacter_sp._Root1280	GE only
Actinomyces_gerencseriae	GE only
Actinomyces_graevenitzii	both
Actinomyces_israelii	GE only
Actinomyces_naeslundii	both
Actinomyces_odontolyticus	GE only
Actinomyces_oris	GE only
Actinomyces_sp._HMSC035G02	GE only
Actinomyces_sp._HMSC075C01	GE only
Actinomyces_sp._HMSC08A09	GE only
Actinomyces_sp._HPA0247	both
Actinomyces_sp._ICM39	GE only
Actinomyces_sp._ICM47	GE only
Actinomyces_sp._ICM54	GE only
Actinomyces_sp._ICM58	GE only
Actinomyces_sp._Marseille.P2825	GE only
Actinomyces_sp._oral_taxon_171	GE only
Actinomyces_sp._oral_taxon_172	GE only
Actinomyces_sp._oral_taxon_175	GE only
Actinomyces_sp._oral_taxon_180	GE only
Actinomyces_sp._oral_taxon_181	GE only
Actinomyces_sp._oral_taxon_448	GE only
Actinomyces_sp._ph3	GE only
Actinomyces_sp._S6.Spd3	GE only
Actinomyces_viscosus	GE only
Aggregatibacter_aphrophilus	GE only
Aggregatibacter_segnis	both
Aggregatibacter_sp._oral_taxon_458	both
Alistipes_putredinis	GE only
Alloprevotella_rava	both
Alloprevotella_tannerae	both
Alloscardovia_omnicolens	GE only
Anaeroglobus_geminatus	GE only
Atopobium_parvulum	GE only
Atopobium_rimae	GE only
Atopobium_sp._BS2	GE only
Atopobium_sp._HMSC064B08	GE only
Atopobium_sp._ICM42b	GE only
Atopobium_sp._oral_taxon_199	GE only
Bacteroidetes_oral_taxon_274	GE only
Bifidobacterium_adolescentis	GE only
Bifidobacterium_dentium	GE only

Bifidobacterium_longum	GE only
Bifidobacterium_sp._12_1_47BFAA	GE only
Bifidobacterium_sp._MSTE12	GE only
Bosea_sp._WAO	GE only
Bulleidia_extructa	GE only
Campylobacter_concisus	both
Campylobacter_rectus	TCGA only
Campylobacter_showae	TCGA only
Campylobacter_sp._10_1_50	GE only
Candida_albicans	GE only
Capnocytophaga_gingivalis	GE only
Capnocytophaga_sp._oral_taxon_329	both
Catonella_morbi	both
Centipeda_periodontii	both
Cloacibacterium_normanense	GE only
Clostridiales_bacterium_KLE1615	GE only
Clostridium_perfringens	GE only
Comamonas_testosteroni	GE only
Corynebacterium_durum	TCGA only
Corynebacterium_kroppenstedtii	GE only
Corynebacterium_matruchotii	GE only
Criibacterium_bergeronii	GE only
Cryptobacterium_curtum	GE only
Delftia_sp._670	GE only
Dialister_invisus	both
Dialister_pneumosintes	both
Dolosigranulum_pigrum	GE only
Eggerthia_catenaformis	GE only
Enhydrobacter_aerosaccus	GE only
Enhydrobacter_sp._H5	GE only
Enterococcus_cecorum	GE only
Escherichia_sp._3_2_53FAA	GE only
Eubacterium_infirmum	TCGA only
Exophiala_oligosperma	TCGA only
Faecalibacterium_prausnitzii	GE only
Filifactor_alocis	both
Fusicatenibacter_saccharivorans	GE only
Fusobacterium_hwasookii	GE only
Fusobacterium_massiliense	GE only
Fusobacterium_nucleatum	GE only
Fusobacterium_periodonticum	GE only
Fusobacterium_sp._CM1	GE only
Fusobacterium_sp._CM21	GE only
Fusobacterium_sp._CM22	GE only
Fusobacterium_sp._HMSC064B11	GE only
Fusobacterium_sp._HMSC064B12	GE only
Fusobacterium_sp._HMSC065F01	GE only
Fusobacterium_sp._OBRC1	GE only
Fusobacterium_sp._oral_taxon_370	GE only
Gemella_haemolysans	both
Gemella_morbillorum	GE only
Gemella_sanguinis	GE only
Gemella_sp._Marseille.P3249	GE only
Gemella_sp._oral_taxon_928	GE only
Granulicatella_adiacens	GE only
Granulicatella_elegans	GE only
Granulicatella_sp._HMSC30F09	GE only
Granulicatella_sp._HMSC31F03	GE only

Haemophilus_aegyptius	GE only
Haemophilus_haemolyticus	GE only
Haemophilus_influenzae	GE only
Haemophilus_parahaemolyticus	GE only
Haemophilus_parainfluenzae	both
Haemophilus_paraphrohaemolyticus	GE only
Haemophilus_sp._C1	GE only
Haemophilus_sp._CCUG_60358	GE only
Haemophilus_sp._CCUG_66565	GE only
Haemophilus_sp._HMSC061E01	GE only
Haemophilus_sp._HMSC066A11	GE only
Haemophilus_sp._HMSC068C11	GE only
Haemophilus_sp._HMSC071C11	GE only
Haemophilus_sp._HMSC073C03	GE only
Haemophilus_sp._HMSC61B11	GE only
Haemophilus_sp._HMSC71H05	GE only
Haemophilus_sp._oral_taxon_851	GE only
Haemophilus_sputorum	GE only
Helicobacter_acinonychis	GE only
Helicobacter_pylori	both
Human_betaherpesvirus_5	both
Human_betaherpesvirus_6B	GE only
Human_betaherpesvirus_7	GE only
Human_gammaherpesvirus_4	manually identified
Johnsonella_ignava	both
Kocuria_sp._HMSC066H03	GE only
Lachnoanaerobaculum_saburreum	both
Lachnoanaerobaculum_sp._ICM7	GE only
Lachnoanaerobaculum_sp._MSX33	GE only
Lachnoanaerobaculum_sp._OBRC5.5	GE only
Lachnospiraceae_bacterium_oral_taxon_082	both
Lachnospiraceae_bacterium_oral_taxon_500	both
Lactobacillus_acidophilus	GE only
Lactobacillus_alimentarius	GE only
Lactobacillus_antri	GE only
Lactobacillus_casei	GE only
Lactobacillus_coleohominis	GE only
Lactobacillus_crispatus	both
Lactobacillus_fermentum	GE only
Lactobacillus_frumenti	GE only
Lactobacillus_gallinarum	GE only
Lactobacillus_gasseri	both
Lactobacillus_gastricus	GE only
Lactobacillus_helveticus	GE only
Lactobacillus_hominis	GE only
Lactobacillus_jensenii	GE only
Lactobacillus_johnsonii	GE only
Lactobacillus_kefiranofaciens	GE only
Lactobacillus_mucosae	GE only
Lactobacillus_oris	GE only
Lactobacillus_panis	GE only
Lactobacillus_paracollinoides	GE only
Lactobacillus_phage_phiadh	GE only
Lactobacillus_reuteri	both
Lactobacillus_salivarius	both
Lactobacillus_sp._HMSC066G01	GE only
Lactobacillus_sp._HMSC24D01	GE only
Lactobacillus_sp._Marseille.P3519	GE only

Lactobacillus_taiwanensis	GE only
Lactobacillus_ultunensis	both
Lactobacillus_vaginalis	both
Lactococcus_lactis	GE only
Lautropia_mirabilis	GE only
Leptotrichia_buccalis	GE only
Leptotrichia_hofstadii	GE only
Leptotrichia_shahii	GE only
Leptotrichia_sp._Marseille.P3007	GE only
Leptotrichia_sp._oral_taxon_212	GE only
Leptotrichia_sp._oral_taxon_215	GE only
Leptotrichia_sp._oral_taxon_225	GE only
Leptotrichia_sp._oral_taxon_847	GE only
Leptotrichia_sp._oral_taxon_879	GE only
Leptotrichia_trevisanii	GE only
Leptotrichia_wadei	both
Limnohabitans_sp._Rim47	GE only
Malassezia_globosa	GE only
Megasphaera_micronuciformis	both
Microbacterium_azadirachtae	GE only
Microbacterium_sp._MRS.1	GE only
Microbacterium_sp._oral_taxon_186	GE only
Micrococcus_aloeverae	GE only
Mitsuokella_sp._oral_taxon_131	both
Mogibacterium_sp._CM50	GE only
Mogibacterium_timidum	GE only
Moraxella_osloensis	GE only
Mycobacterium_sp._1554424.7	GE only
Neisseria_mucosa	GE only
Neisseria_sp._HMSC061B04	GE only
Neisseria_sp._HMSC063B05	GE only
Neisseria_sp._HMSC065C04	GE only
Neisseria_sp._HMSC066H01	GE only
Neisseria_sp._HMSC073G10	GE only
Neisseria_sp._HMSC075C12	GE only
Neisseria_sp._HMSC078C12	GE only
Neisseria_sp._HMSC15G01	GE only
Neisseria_sp._HMSC31F04	GE only
Neisseria_sp._oral_taxon_014	GE only
Olsenella_profusa	GE only
Olsenella_uli	both
Oribacterium_parvum	TCGA only
Oribacterium_sinus	GE only
Oribacterium_sp._oral_taxon_078	both
Oribacterium_sp._oral_taxon_108	GE only
Parabacteroides_merdae	GE only
Paracoccus_sp._228	GE only
Paracoccus_yeei	GE only
Parascardovia_denticolens	GE only
Parvimonas_micra	both
Parvimonas_sp._oral_taxon_110	GE only
Parvimonas_sp._oral_taxon_393	GE only
Peptoanaerobacter_stomatis	both
Peptoniphilus_indolicus	GE only
Peptoniphilus_lacrimalis	GE only
Peptoniphilus_sp._BV3C26	GE only
Peptostreptococcaceae_bacterium_oral_taxon_113	GE only
Peptostreptococcus_anaerobius	GE only

Peptostreptococcus_sp._MV1	GE only
Peptostreptococcus_stomatis	both
Porphyromonadaceae_bacterium_KA00676	GE only
Porphyromonas_endodontalis	both
Porphyromonas_gingivalis	both
Porphyromonas_sp._KLE_1280	GE only
Porphyromonas_sp._oral_taxon_279	GE only
Prevotella_amnii	GE only
Prevotella_aurantiaca	both
Prevotella_baroniae	both
Prevotella_bergensis	GE only
Prevotella_bivia	GE only
Prevotella_buccae	both
Prevotella_buccalis	GE only
Prevotella_conceptionensis	GE only
Prevotella_copri	GE only
Prevotella_corporis	GE only
Prevotella_dentalis	GE only
Prevotella_denticola	both
Prevotella_disiens	both
Prevotella_enoeca	both
Prevotella_fusca	both
Prevotella_histicola	both
Prevotella_ihumii	GE only
Prevotella_intermedia	both
Prevotella_loescheii	TCGA only
Prevotella_maculosa	both
Prevotella_marshii	both
Prevotella_melaninogenica	both
Prevotella_multiformis	both
Prevotella_multisaccharivorax	both
Prevotella_nanceiensis	both
Prevotella_nigrescens	both
Prevotella_oris	both
Prevotella_oulorum	both
Prevotella_pallens	both
Prevotella_phocaeensis	GE only
Prevotella_pleuritidis	both
Prevotella_saccharolytica	GE only
Prevotella_salivae	both
Prevotella_scopos	both
Prevotella_shahii	GE only
Prevotella_sp._BV3P1	GE only
Prevotella_sp._C561	both
Prevotella_sp._F0091	both
Prevotella_sp._HJM029	both
Prevotella_sp._HMSC069G02	both
Prevotella_sp._HMSC073D09	GE only
Prevotella_sp._HMSC077E08	GE only
Prevotella_sp._HMSC077E09	GE only
Prevotella_sp._HUN102	GE only
Prevotella_sp._ICM33	both
Prevotella_sp._MSX73	GE only
Prevotella_sp._oral_taxon_299	GE only
Prevotella_sp._oral_taxon_306	both
Prevotella_sp._oral_taxon_317	GE only
Prevotella_sp._oral_taxon_472	GE only
Prevotella_sp._oral_taxon_473	TCGA only

Prevotella_sp._S7.1.8	GE only
Prevotella_timonensis	GE only
Prevotella_veroralis	both
Prevotellaceae_bacterium_Marseille.P2826	TCGA only
Propionibacterium_acidifaciens	GE only
Pseudomonas_syringae_pv._coryli	GE only
Pseudomonas_veronii	GE only
Psychrobacter_sp._SHUES1	GE only
Rhodococcus_sp._1139	GE only
Romboutsia_timonensis	GE only
Roseburia_intestinalis	GE only
Roseburia_inulinivorans	GE only
Rothia_mucilaginosa	GE only
Rothia_sp._HMSC061C12	GE only
Rothia_sp._HMSC061D12	GE only
Rothia_sp._HMSC061E04	GE only
Rothia_sp._HMSC062F03	GE only
Rothia_sp._HMSC062H08	GE only
Rothia_sp._HMSC064F07	GE only
Rothia_sp._HMSC065B04	GE only
Rothia_sp._HMSC065C03	GE only
Rothia_sp._HMSC065C12	GE only
Rothia_sp._HMSC066G02	GE only
Rothia_sp._HMSC066G07	GE only
Rothia_sp._HMSC068E02	GE only
Rothia_sp._HMSC068F09	GE only
Rothia_sp._HMSC069C04	GE only
Rothia_sp._HMSC069C10	GE only
Rothia_sp._HMSC069D01	GE only
Rothia_sp._HMSC071B01	GE only
Rothia_sp._HMSC071C12	GE only
Rothia_sp._HMSC072B03	GE only
Rothia_sp._HMSC072B04	GE only
Rothia_sp._HMSC072E10	GE only
Rothia_sp._HMSC075F09	GE only
Rothia_sp._HMSC076D04	GE only
Rothia_sp._HMSC078H08	GE only
Ruminococcus_faecis	GE only
Selenomonas_flueggei	both
Selenomonas_infelix	both
Selenomonas_sp._CM52	both
Selenomonas_sp._oral_taxon_126	both
Selenomonas_sp._oral_taxon_136	both
Selenomonas_sp._oral_taxon_138	TCGA only
Selenomonas_sp._oral_taxon_149	both
Selenomonas_sp._oral_taxon_478	GE only
Selenomonas_sp._oral_taxon_892	both
Selenomonas_sp._oral_taxon_920	both
Selenomonas_sputigena	both
Shuttleworthia_satelles	GE only
Shuttleworthia_sp._MSX8B	GE only
Slackia_exigua	both
Slackia_sp._CM382	both
Solobacterium_moorei	both
Sphingomonas_melonis	GE only
Staphylococcus_warneri	GE only
Stomatobaculum_longum	both
Streptococcus_anginosus	GE only

Streptococcus_australis	GE only
Streptococcus_constellatus	GE only
Streptococcus_cristatus	GE only
Streptococcus_gordonii	GE only
Streptococcus_infantis	GE only
Streptococcus_intermedius	GE only
Streptococcus_lutetiensis	GE only
Streptococcus_macedonicus	GE only
Streptococcus_massiliensis	GE only
Streptococcus_mitis	both
Streptococcus_mutans	GE only
Streptococcus_oralis	both
Streptococcus_parasanguinis	GE only
Streptococcus_peroris	GE only
Streptococcus_phage_EJ.1	GE only
Streptococcus_pneumoniae	GE only
Streptococcus_pseudopneumoniae	GE only
Streptococcus_salivarius	GE only
Streptococcus_sanguinis	GE only
Streptococcus_sinensis	GE only
Streptococcus_sp._1004_SSPC	GE only
Streptococcus_sp._1171_SSPC	GE only
Streptococcus_sp._2_1_36FAA	GE only
Streptococcus_sp._263_SSPC	GE only
Streptococcus_sp._343_SSPC	GE only
Streptococcus_sp._400_SSPC	GE only
Streptococcus_sp._449_SSPC	GE only
Streptococcus_sp._A12	GE only
Streptococcus_sp._ACS2	GE only
Streptococcus_sp._AS20	GE only
Streptococcus_sp._BS29a	GE only
Streptococcus_sp._C150	GE only
Streptococcus_sp._C300	GE only
Streptococcus_sp._CCH5.D3	GE only
Streptococcus_sp._CCH8.C6	GE only
Streptococcus_sp._CCH8.G7	GE only
Streptococcus_sp._CCH8.H5	GE only
Streptococcus_sp._CCUG_49591	GE only
Streptococcus_sp._CM6	GE only
Streptococcus_sp._CM7	GE only
Streptococcus_sp._DD04	GE only
Streptococcus_sp._DD10	GE only
Streptococcus_sp._F0441	GE only
Streptococcus_sp._F0442	GE only
Streptococcus_sp._FDAARGOS_146	GE only
Streptococcus_sp._GMD3S	GE only
Streptococcus_sp._GMD4S	GE only
Streptococcus_sp._GMD5S	GE only
Streptococcus_sp._HMSC034A12	GE only
Streptococcus_sp._HMSC034B03	GE only
Streptococcus_sp._HMSC034B05	GE only
Streptococcus_sp._HMSC034E03	GE only
Streptococcus_sp._HMSC034E12	GE only
Streptococcus_sp._HMSC056C01	GE only
Streptococcus_sp._HMSC057E02	GE only
Streptococcus_sp._HMSC057G03	GE only
Streptococcus_sp._HMSC061D01	GE only
Streptococcus_sp._HMSC061D10	GE only

Streptococcus_sp._HMSC061E03	GE only
Streptococcus_sp._HMSC062D07	GE only
Streptococcus_sp._HMSC062H02	GE only
Streptococcus_sp._HMSC063B03	GE only
Streptococcus_sp._HMSC064D12	GE only
Streptococcus_sp._HMSC064H09	GE only
Streptococcus_sp._HMSC065C01	GE only
Streptococcus_sp._HMSC065E03	GE only
Streptococcus_sp._HMSC065H07	GE only
Streptococcus_sp._HMSC066E07	GE only
Streptococcus_sp._HMSC066F01	GE only
Streptococcus_sp._HMSC067A03	GE only
Streptococcus_sp._HMSC067H01	GE only
Streptococcus_sp._HMSC068F04	GE only
Streptococcus_sp._HMSC070B10	GE only
Streptococcus_sp._HMSC071D03	GE only
Streptococcus_sp._HMSC072C09	GE only
Streptococcus_sp._HMSC072D03	GE only
Streptococcus_sp._HMSC072D05	GE only
Streptococcus_sp._HMSC072D07	GE only
Streptococcus_sp._HMSC072G04	GE only
Streptococcus_sp._HMSC073A12	GE only
Streptococcus_sp._HMSC073D05	GE only
Streptococcus_sp._HMSC073F11	GE only
Streptococcus_sp._HMSC074B11	GE only
Streptococcus_sp._HMSC074F05	GE only
Streptococcus_sp._HMSC076C08	GE only
Streptococcus_sp._HMSC076C09	GE only
Streptococcus_sp._HMSC077D04	GE only
Streptococcus_sp._HMSC077F03	GE only
Streptococcus_sp._HMSC078D09	GE only
Streptococcus_sp._HMSC078H03	GE only
Streptococcus_sp._HMSC078H12	GE only
Streptococcus_sp._HMSC10A01	GE only
Streptococcus_sp._HMSC10E12	GE only
Streptococcus_sp._HMSC34B10	GE only
Streptococcus_sp._HMSC36C04	GE only
Streptococcus_sp._HPH0090	GE only
Streptococcus_sp._HSISS1	GE only
Streptococcus_sp._HSISS3	GE only
Streptococcus_sp._I.G2	GE only
Streptococcus_sp._I.P16	GE only
Streptococcus_sp._M143	GE only
Streptococcus_sp._M334	GE only
Streptococcus_sp._OBRC6	GE only
Streptococcus_sp._oral_taxon_056	GE only
Streptococcus_sp._oral_taxon_058	GE only
Streptococcus_sp._oral_taxon_064	GE only
Streptococcus_sp._oral_taxon_071	both
Streptococcus_sp._oral_taxon_431	GE only
Streptococcus_sp._SK140	GE only
Streptococcus_sp._SK643	GE only
Streptococcus_sp._SR1	GE only
Streptococcus_sp._SR4	GE only
Streptococcus_thermophilus	GE only
Streptococcus_timonensis	GE only
Streptococcus_vestibularis	GE only
Tannerella_forsythia	both

Tannerella_sp._oral_taxon_HOT.286	GE only
Tepidimonas_fonticaldi	GE only
Tepidimonas_taiwanensis	GE only
Thermosipho_affectus	GE only
Treponema_denticola	both
Treponema_lecithinolyticum	TCGA only
Treponema_maltophilum	both
Treponema_medium	TCGA only
Treponema_socranskii	TCGA only
Treponema_sp._OMZ_838	TCGA only
Treponema_vincentii	TCGA only
Tyzzerella_nexilis	GE only
Veillonella_atypica	GE only
Veillonella_dispar	both
Veillonella_parvula	both
Veillonella_sp._3_1_44	both
Veillonella_sp._6_1_27	both
Veillonella_sp._ACP1	GE only
Veillonella_sp._AS16	GE only
Veillonella_sp._HPA0037	GE only
Veillonella_sp._ICM51a	GE only
Veillonella_sp._oral_taxon_158	both
Veillonella_tobetsuensis	GE only
X.Eubacterium._brachy	GE only
X.Eubacterium._eligens	GE only
X.Eubacterium._hallii	GE only
X.Eubacterium._infirmum	GE only
X.Eubacterium._nodatum	GE only
X.Eubacterium._rectale	GE only
X.Eubacterium._saphenum	GE only
X.Eubacterium._sulci	GE only
X.Hallella._seregens	GE only
X.Propionibacterium._namnetense	GE only
Xanthomonas_campestris	GE only

‘GE only’, identified as tissue-resident from Genomics England 100,000 Genomes Project data; ‘TCGA only’, identified as tissue-resident from The Cancer Genome Atlas data only; ‘both’, identified as tissue-resident from both 100,000 Genomes Project and TCGA cohorts; ‘manually identified’, identified as tissue-resident due to accepted non-contaminant status.

Online Resource Table 2. List of genera present on study include list following decontamination process.

Abiotrophia
Achromobacter
Acidovorax
Acinetobacter
Actinomyces
Aggregatibacter
Alistipes
Alloprevotella
Alloscardovia
Anaeroglobus
Atopobium
Bacteroidetes
Bifidobacterium
Bosea
Bulleidia
Campylobacter
Candida
Capnocytophaga
Catonella
Centipeda
Cloacibacterium
Clostridiales
Clostridium
Comamonas
Corynebacterium
Criibacterium
Cryptobacterium
Delftia
Dialister
Dolosigranulum
Eggerthia
Enhydrobacter
Enterococcus
Escherichia
Eubacterium
Exophiala
Faecalibacterium
Filifactor
Fusicatenibacter
Fusobacterium
Gemella
Granulicatella
Haemophilus
Helicobacter
Cytomegalovirus
Roseolovirus
Lymphocryptovirus
Johnsonella
Kocuria
Lachnoanaerobaculum
Lachnospiraceae
Lactobacillus
Lactococcus
Lautropia
Leptotrichia
Limnohabitans
Malassezia

Megasphaera
Microbacterium
Micrococcus
Mitsuokella
Mogibacterium
Moraxella
Mycobacterium
Neisseria
Olsenella
Oribacterium
Parabacteroides
Paracoccus
Parascardovia
Parvimonas
Peptoanaerobacter
Peptoniphilus
Peptostreptococcaceae
Peptostreptococcus
Porphyromonadaceae
Porphyromonas
Prevotella
Prevotellaceae
Propionibacterium
Pseudomonas
Psychrobacter
Rhodococcus
Romboutsia
Roseburia
Rothia
Ruminococcus
Selenomonas
Shuttleworthia
Slackia
Solobacterium
Sphingomonas
Staphylococcus
Stomatobaculum
Streptococcus
Tannerella
Tepidimonas
Thermosipho
Treponema
Tyzzerella
Veillonella
X.Eubacterium.
X.Hallella.
X.Propionibacterium.
Xanthomonas

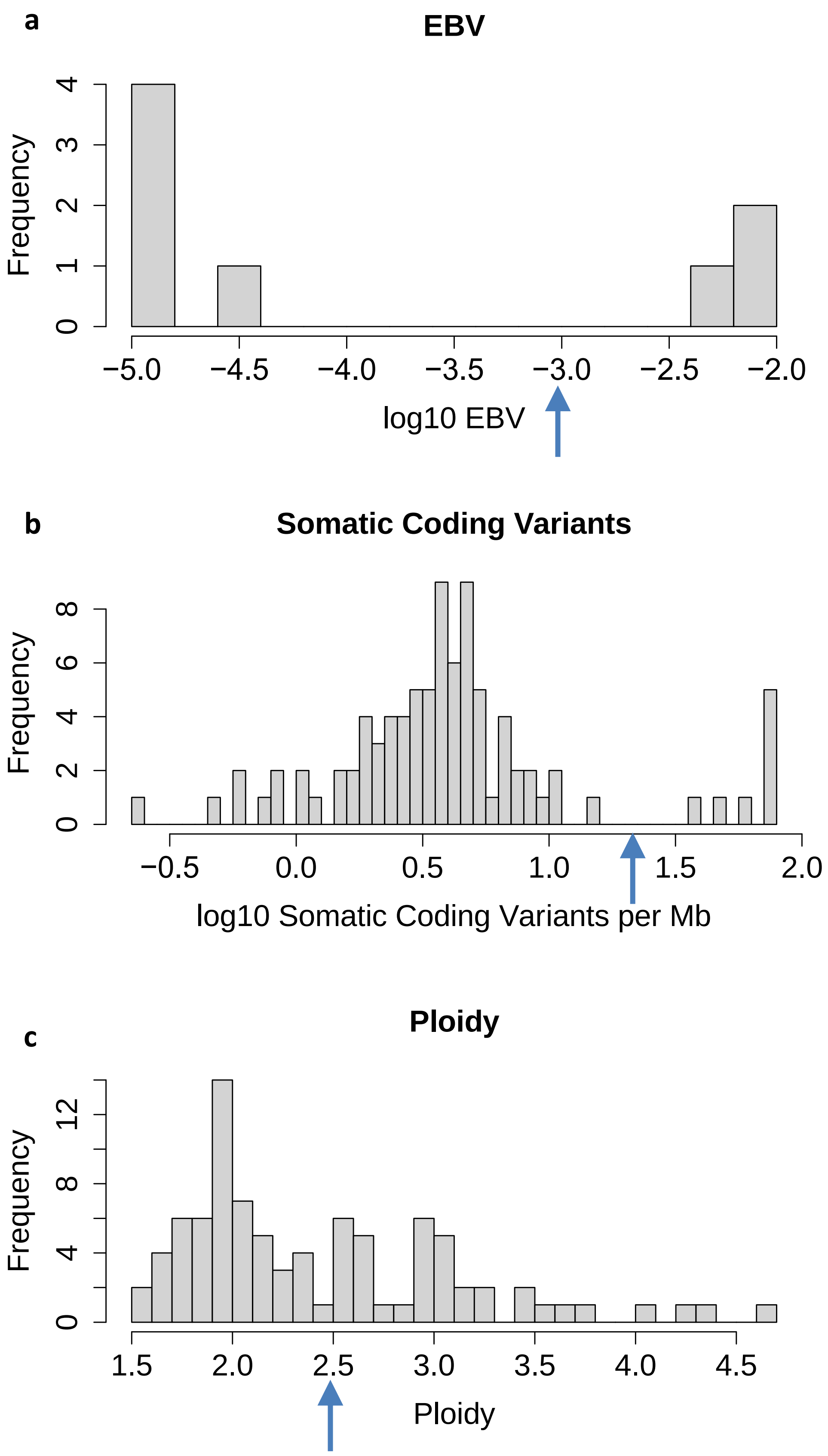

Online Resource Figure 1. Histograms of (a) non-zero EBV counts (log10), (b) somatic coding variants (log10), and (c) total ploidy in 89 gastric and junctional adenocarcinomas within the 100,000 Genomes Project. Blue arrows represent thresholds used to define binary categories as described in main text.

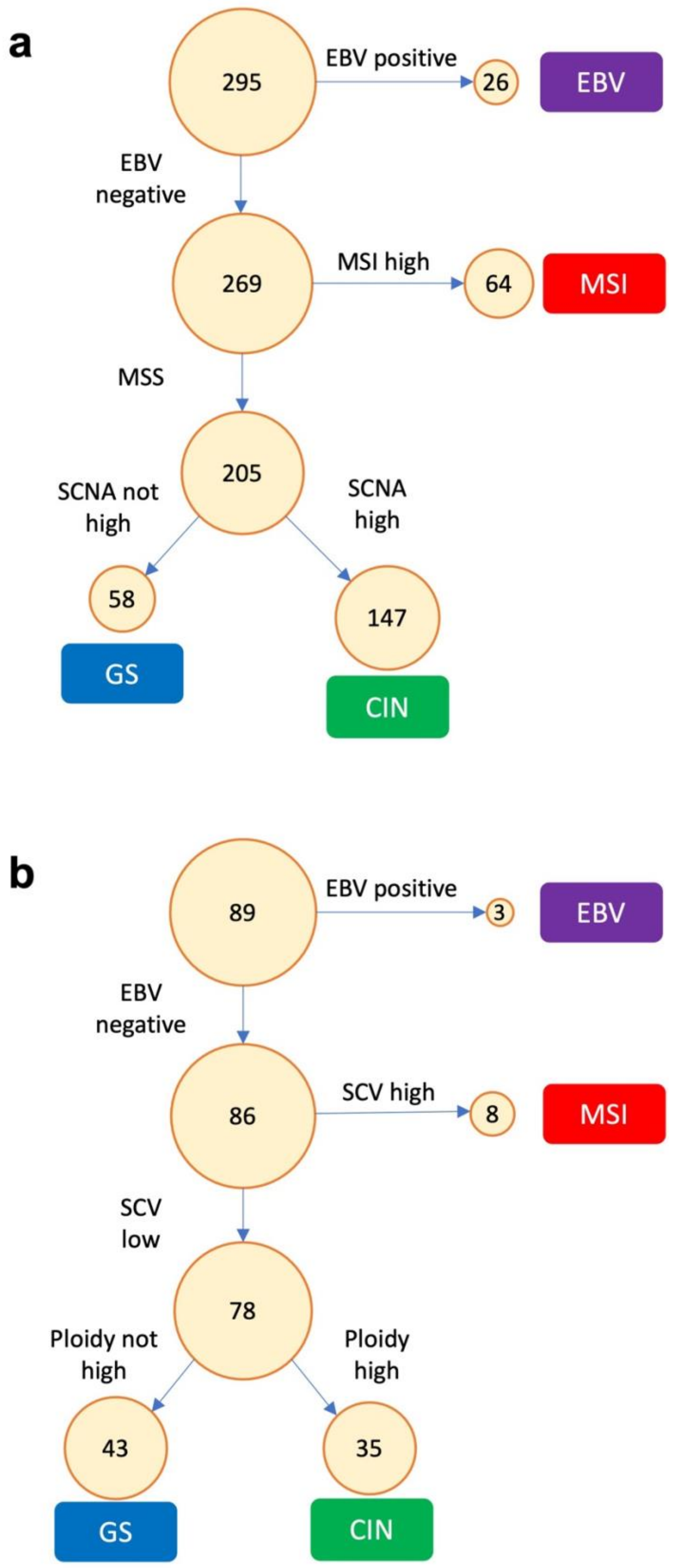

Online Resource Figure 2. Classification of TCGA molecular subtypes (a) method used to classify samples within TCGA cohort, as previously described by Thorsson *et al*., (b) method used to categorist samples from the 100,000 Genomes Project. Threshold values (as demonstrated in supplementary figure 1) were used to infer TCGA molecular subtype for 100,000 Genomes Project samples. Samples were classed as EBV subtype if the EBV sequencing count was greater than or equal to the EBV threshold value. Samples not assigned to the EBV subtype, with SCVs greater than or equal to the SCV threshold value were assigned to the MSI subtype. Remaining samples were assigned to the GS subtype if the ploidy value was below the ploidy threshold value or the CIN subtype if the DNA ploidy value was greater than or equal to the DNA ploidy threshold value. CIN, chromosomal instability; EBV, Epstein-Barr virus; GS genomically stable; MSI; MSS, microsatellite stable; microsatellite instability; SCNA, somatic copy-number alterations; SCV, somatic coding variants

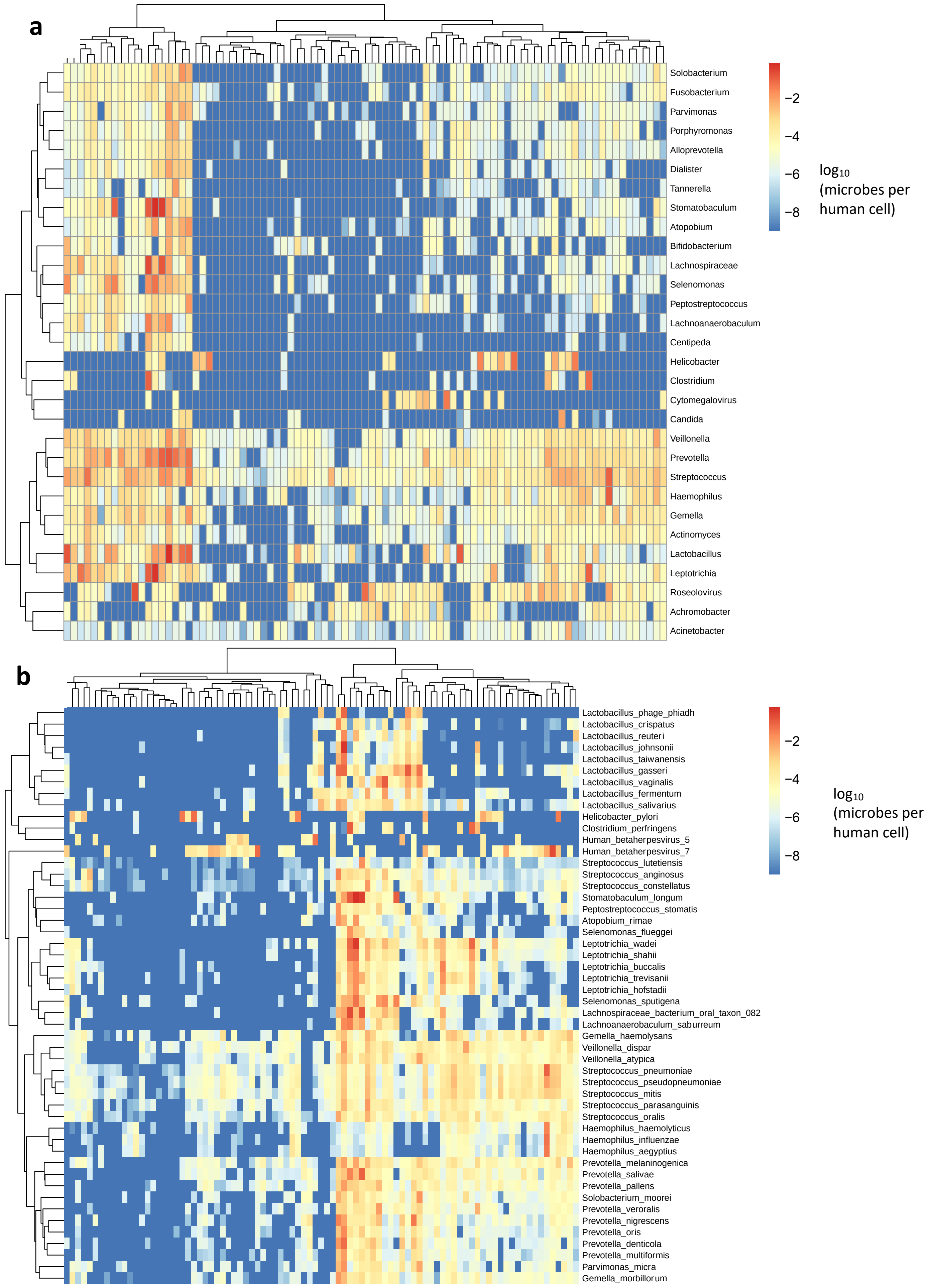

Online Resource Figure 3. Heatmaps of microbes per human cell in 89 gastric cancer samples from the 100,000 Genomes Project cohort, showing (a) 30 most abundant genera and (b) 50 most abundant species. Cells are coloured according to $\log_{10}$(microbes per human cell) of taxa from red (highest) to blue (lowest). Branching on the left signifies clustering genera; branching at the top represents samples with similar compositions of genera.

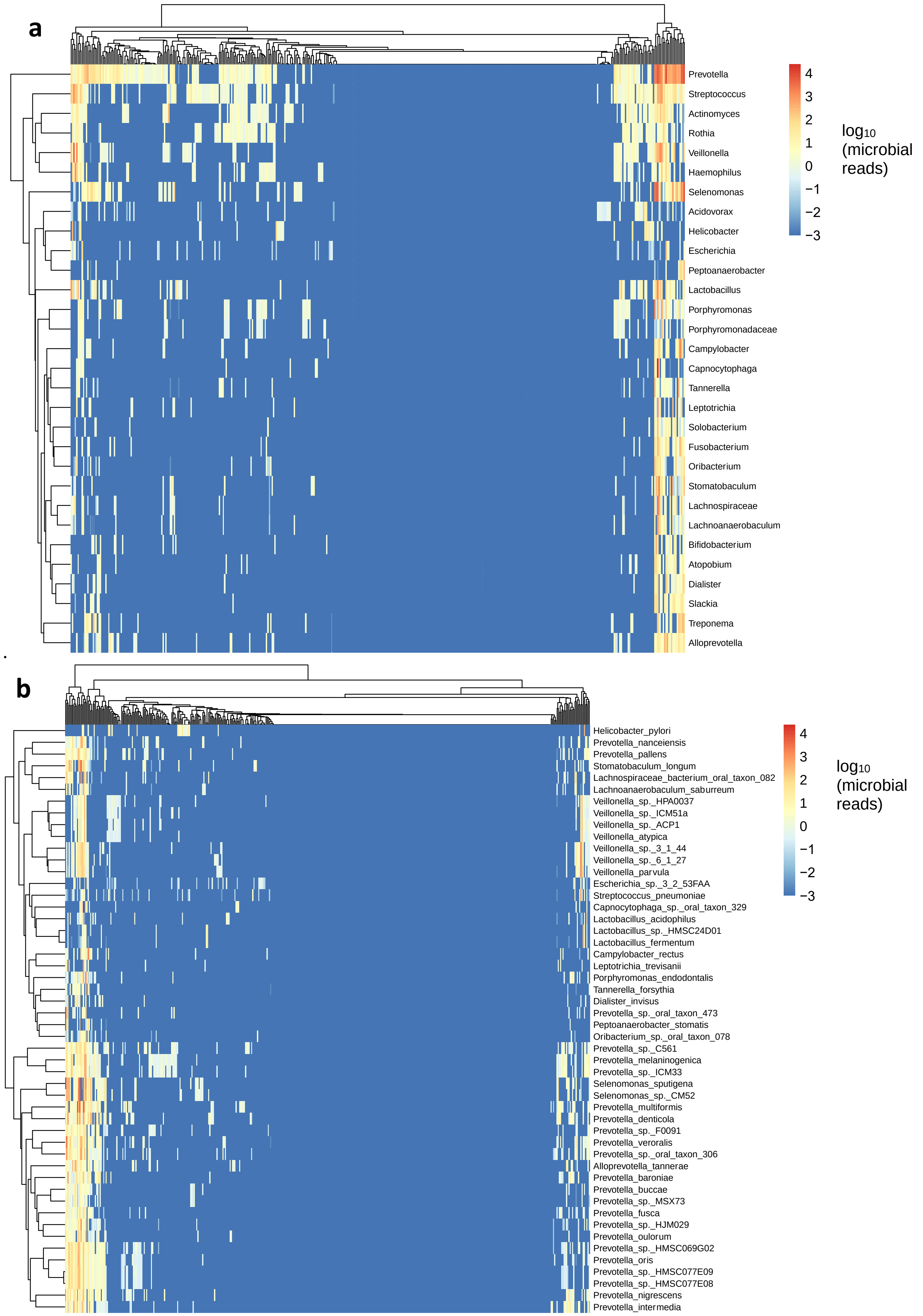

Online Resource Figure 4. Heatmaps of microbial abundance in 440 gastric cancer samples from the TCGA cohort, showing (a) 30 most abundant genera and (b) 50 most abundant species. Cells are coloured according to $\log_{10}$(microbial reads) of taxa from red (highest) to blue (lowest). Branching on the left signifies clustering genera; branching at the top represents samples with similar compositions of genera.
